# Robotic Versus Laparoscopic Pancreaticoduodenectomy: An Up-To-Date System Review and Meta-Analysis

**DOI:** 10.3389/fonc.2022.834382

**Published:** 2022-02-25

**Authors:** Lanwei Ouyang, Jia Zhang, Qingbo Feng, Zhiguang Zhang, Hexing Ma, Guodong Zhang

**Affiliations:** ^1^ Department of Thoracic Surgery, The 3rd Affiliated Hospital Of Chengdu Medical College, Pidu District People’s Hospital, Chengdu, China; ^2^ Department of Breast Surgery, West China Hospital of Sichuan University, Chengdu, China; ^3^ Department of Liver Surgery and Liver Transplantation Centre, West China Hospital, Sichuan University, Chengdu, China; ^4^ Department of General Surgery, The Affiliated Hospital of Yangzhou University, Yangzhou University, Yangzhou, China

**Keywords:** minimally invasive surgery, robotic pancreaticoduodenectomy, laparoscopic pancreaticoduodenectomy, Da Vinci, meta-analysis

## Abstract

**Background:**

Although minimally invasive pancreaticoduodenectomy has gained worldwide interest, there are limited comparative studies between two minimally invasive pancreaticoduodenectomy techniques. This meta-analysis aimed to compare the safety and efficacy of robotic and laparoscopic pancreaticoduodenectomy (LPD), especially the difference in the perioperative and short-term oncological outcomes.

**Methods:**

PubMed, China National Knowledge Infrastructure (CNKI), Wanfang Data, Web of Science, and EMBASE were searched based on a defined search strategy to identify eligible studies before July 2021. Data on operative times, blood loss, overall morbidity, major complications, vascular resection, blood transfusion, postoperative pancreatic fistula (POPF), delayed gastric emptying (DGE), conversion rate, reoperation, length of hospital stay (LOS), and lymph node dissection were subjected to meta-analysis.

**Results:**

Overall, the final analysis included 9 retrospective studies comprising 3,732 patients; 1,149 (30.79%) underwent robotic pancreaticoduodenectomy (RPD), and 2,583 (69.21%) underwent LPD. The present meta-analysis revealed nonsignificant differences in operative times, overall morbidity, major complications, blood transfusion, POPF, DGE, reoperation, and LOS. Alternatively, compared with LPD, RPD was associated with less blood loss (p = 0.002), less conversion rate (p < 0.00001), less vascular resection (p = 0.0006), and more retrieved lymph nodes (p = 0.01).

**Conclusion:**

RPD is at least equivalent to LPD with respect to the incidence of complication, incidence and severity of DGE, and reoperation and length of hospital stay. Compared with LPD, RPD seems to be associated with less blood loss, lower conversion rate, less vascular resection, and more retrieved lymph nodes.

**Systematic Review Registration:**

https://www.crd.york.ac.uk/PROSPERO/#recordDetails, identifier CRD2021274057

## Introduction

Pancreaticoduodenectomy is a very complex procedure and is considered to be the standard of surgical treatment for both benign and malignant cancer in the periampullary region and pancreatic head (1). Gagner et al. reported the first successful laparoscopic pancreaticoduodenectomy (LPD) in 1994 (2). Almost a decade later, the development of innovative robotic platforms has later opened a new horizon for surgical treatment of pancreatic cancer, with Giulianotti et al. performing the first robotic pancreaticoduodenectomy (RPD) in Italy in 2003 (3). RPD and LPD are both minimally invasive treatments for pancreatic and periampullary malignancies and some benign diseases, but only 285 reported LPD cases have been reported as of 2011, and the safety and feasibility of RPD and LPD remain controversial (4).

To date, although several studies reporting on minimally invasive pancreaticoduodenectomy have been reported, a few studies compare one minimally invasive technique to open (5–7). Pooled data from these retrospective studies have shown that RPD was associated with less blood loss and shorter hospital stay as compared with open pancreaticoduodenectomy. At present, evidence comparing the benefits of RPD and LPD is limited. Although one meta-analysis has compared the safety and feasibility of RPD with LPD, the meta-analysis comprised only 6 studies, and most of them are of small sample size and low quality and have no randomized controlled trial (RCT), which limited them to deduce objective conclusion (8).

In the present study, we performed a meta-analysis of updated data from currently available studies to compare perioperative outcomes and short-term oncological outcomes after RPD and LPD.

## Methods

### Search Strategy and Study Selection

A systematic review was performed and adhered to the Preferred Reporting Items for Systematic Reviews and Meta-Analyses (PRISMA) guidelines, and the study protocol was registered at PROSPERO with the registration number CRD2021274057 (9). Given that the first RPD was reported in 2003, a systematic literature search for published studies that investigated RPD versus LPD was performed in PubMed, EMBASE, Web of Science, CNKI, and Wanfang Data, from January 1, 2003, to July 25, 2021, by two authors (LO and JZ). The combinations of the following keywords were used: RPD, LPD, Da Vinci surgery, and minimally invasive surgery. In order to gain additional studies, the references of eligible studies were manually searched.

### Inclusion and Exclusion Criteria

All titles and abstracts were screened and identified eligible studies according to the following criteria by two investigators (LO and JZ) independently.

Articles meeting these criteria will be included in the analysis: 1) participants, patients’ age >18. 2) Types of interventions: RPD and LPD. 3) Types of studies: retrospective studies, cohort studies, case–control studies, and RCTs. 4) Data available on interesting perioperative and oncological outcomes. 5) Studies published in English or Chinese.

Exclusion criteria were as follows: 1) data that were incomplete and 2) editorials, abstracts, letters, case reports, and expert opinions.

### Data Extraction and Quality Assessment

The original data from all candidate articles were individually assessed and extracted by two reviewers (LO and JZ) by using a unified datasheet, and any ambiguity was resolved by a third researcher (QF). The major data extraction includes the following: name of the first or corresponding author, study design, publication year, country, sample size, mean age, gender, body mass index, operative times, bleeding, overall complications, major complications, tumor size, number of retrieved lymph nodes (LNs), blood transfusion, vascular resection, blood transfusion, postoperative pancreatic fistula (POPF), delayed gastric emptying (DGE), conversion rate, and reoperation. The Newcastle–Ottawa Scale (NOS) was adopted to assess the quality of the eligible studies (10). Every included study was independently evaluated by two authors (LO and JZ), and a NOS score ≥6 is considered as being of high quality.

### Statistical Analysis

The Review Manager 5.3 software was used for statistical analyses. 95% CI and mean difference (MD) were used for continuous data, while dichotomous data used odds ratio (OR). The method originally described by Hozo et al. was used to convert medians with ranges into means with SDs (11). Begg’s funnel plot and Egger’s test were used to assess potential publication bias. Statistical heterogeneity was quantified using Higgin’s I^2^ index. A fixed-effects model (FEM) was adopted when heterogeneity is low or moderate (I^2^ < 50%), while heterogeneity is high (I^2^ ≥ 50%) when a random-effects model (REM) was used.

## Results

### Characteristics of the Included Studies

**Figure 1:**
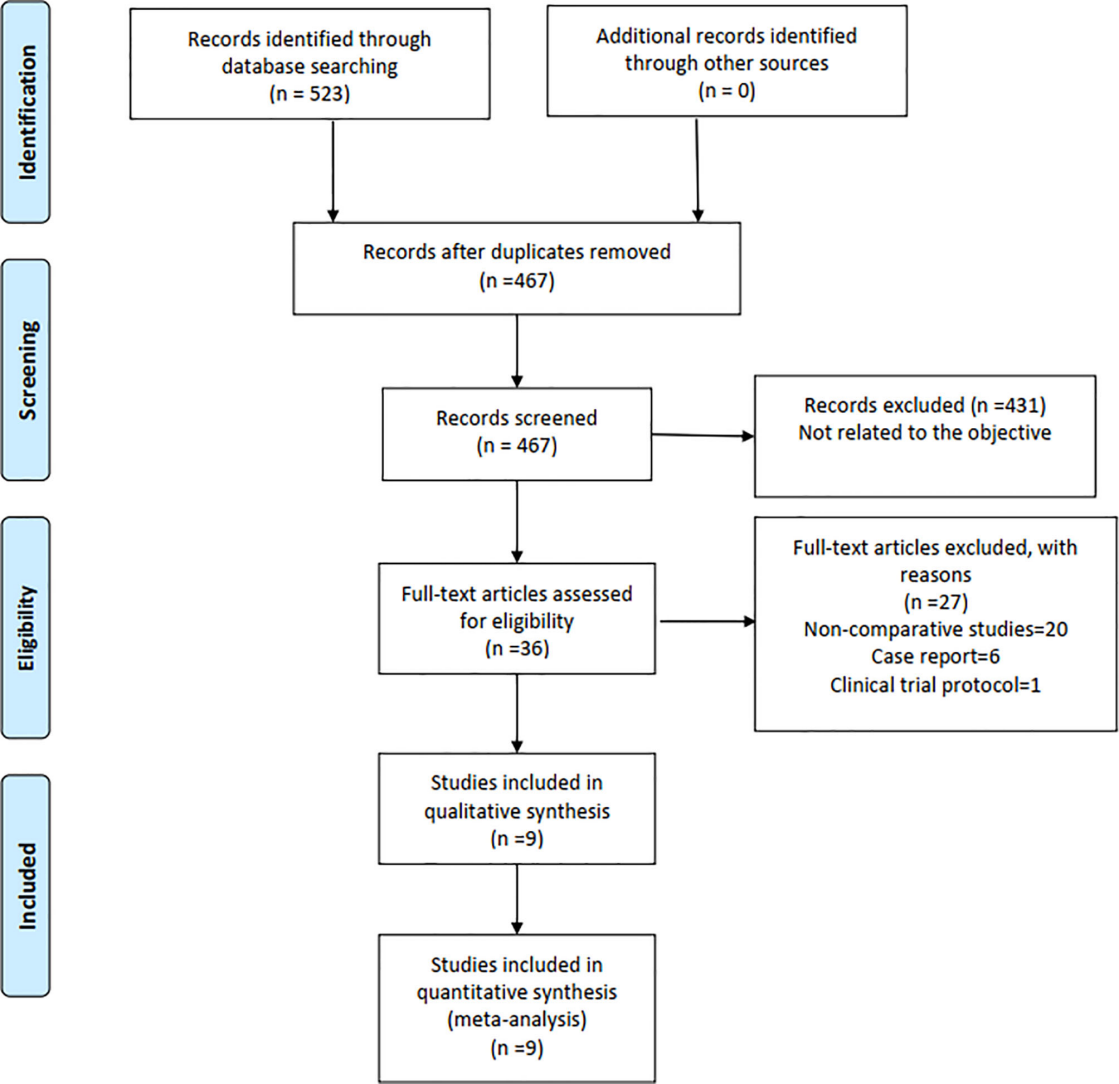
Flowchart of study identification and selection.

Finally, a total of 523 relevant English and Chinese publications from various electronic databases were yielded. Finally, according to the inclusion criteria, 9 retrospective studies (12–20) comparing RPD and LPD in a total of 3,732 patients (1,149 and 2,583 patients underwent RPD and LPD, respectively) were included for further analysis. A flow diagram of our analysis protocol is shown in **Figure 1**. The general information and summary of NOS scores of all the included studies are given in **Table 1**.

**Table 1:** Characteristics of included studies.

Author (year)	Type of study	Period	Country	Patients (n)		Age (years)		Gender (M/F)		BMI		NOS
				RPD	LPD	RPD	LPD	RPD	LPD	RPD	LPD	
Liu (2016)	Retrospective	2015–2016	China	27	25	57.16 ± 8.56	60.54 ± 18.25	14/13	12/13	NA	NA	8
Nassour (2017)	Retrospective	2014–2015	USA	193	235	63.5 ± 11.9	63.4 ± 11.6	101/92	129/106	27.8 ± 5.3	27.6 ± 6.6	7
Zimmerman (2017)	Retrospective	2014–2015	USA	211	280	66 (68–72)	64 (57–72)	109/102	159/121	27.3 (23.8–30.9)	26.9 (23.5–30.9)	7
Nassour (2018)	Retrospective	2010–2013	USA	165	1458	66.5	66.3	81/84	756/702	NA	NA	7
Xourafas (2018)	Retrospective	2014–2016	USA	409	418	64 (18–88)	63 (19–87)	216/193	233/185	27.5 (19–51)	27.6 (16–67)	7
Zhang (2018)	Retrospective	2013–2017	China	20	20	68 (50–78)	64 (42–76)	12/8	11/9	24.8 ± 2.5	24.0 ± 3.5	6
Goh (2018)	Retrospective	2014–2017	Singapore	10	20	70 (53–78)	62.5 (24–79)	5/5	16/4	21.3 (18–27.6)	20.6 (14–26)	7
Oosten (2020)	PSM	2011–2019	USA	90	90	67 (60–73)	67 (58–75)	NA	NA	26 (23–29)	25 (22–29)	8
Xu (2021)	Retrospective	2016–2019	China	24	37	64.0 ± 9.4	61.0 ± 9.6	11/11	20/17	20.0 (18.8–21.9)	20.4 (19.2–21.4)	8

RPD, robotic pancreaticoduodenectomy; LPD, laparoscopic pancreaticoduodenectomy; M/F, male/female; BMI, body mass index; NA, not available; NOS, Newcastle–Ottawa Scale; PSM, propensity score matching.

### Operative Outcomes

#### Operative Time

**Figure 2:**
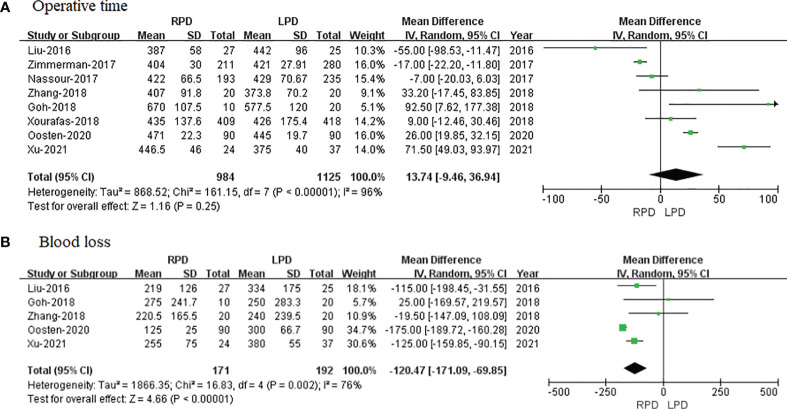
Forest plot of comparison of RPD versus LPD for operative outcomes. **(A)** Forest plot for operative time. **(B)** Forest plot for blood loss. RPD, robotic pancreaticoduodenectomy; LPD, laparoscopic pancreaticoduodenectomy.

Eight of the included 8 studies that encompassed 2,109 patients (984 and 1,125 underwent RPD and LPD, respectively) reported operative time. The present meta-analysis showed that RPD has a similar operative time as compared with the LPD group (MD = 13.74 min; 95% CI −9.46 to 36.94; p = 0.25). Heterogeneity was high (I^2^ = 96%) and analyzed in the REM (**Figure 2A**).

#### Blood Loss

Five studies with a total of 363 patients had reported the bleeding volume. A meta-analysis of 5 studies indicated that RPD had less blood loss as compared to LPD (MD = −120.47 ml; 95% CI −171.09 to −69.85; p < 0.00001). Heterogeneity was high (I^2^ = 76%) and analyzed in the REM (**Figure 2B**).

### Postoperative Outcomes

#### Number of Retrieved Lymph Node

**Figure 3:**
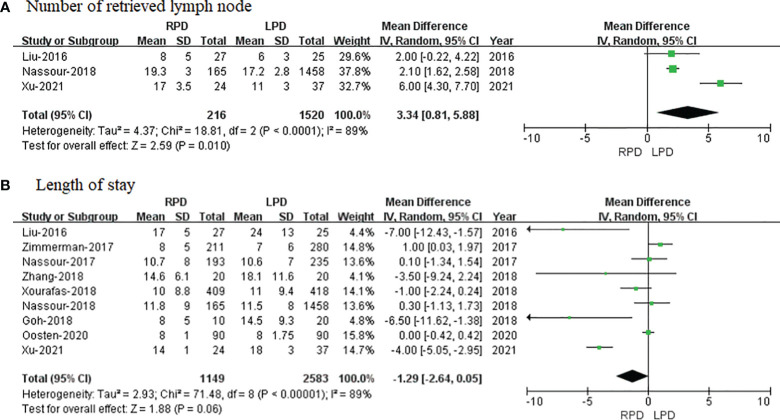
Forest plot of comparison of RPD versus LPD for postoperative outcomes. **(A)** Forest plot for a number of retrieved lymph nodes. **(B)** Forest plot for the length of stay. RPD, robotic pancreaticoduodenectomy; LPD, laparoscopic pancreaticoduodenectomy.

The number of retrieved LN data was available in 3 studies. Noticeably, the meta-analysis suggested that RPD present markedly more retrieved LN than the LPD group (OR = 3.34; 95% CI 0.81 to 5.88; p = 0.001). Heterogeneity was high (I^2^ = 89%) and analyzed in the REM (**Figure 3A**).

#### Length of Stay

Length of stay data were available in 9 studies. The meta-analysis showed no difference in hospital stay between the two groups (MD = − 1.29; 95% CI – 2.64 to 0.05; p = 0.06), with high heterogeneity (I^2^ = 89%) in the REM (**Figure 3B**).

#### Overall Complications

**Figure 4:**
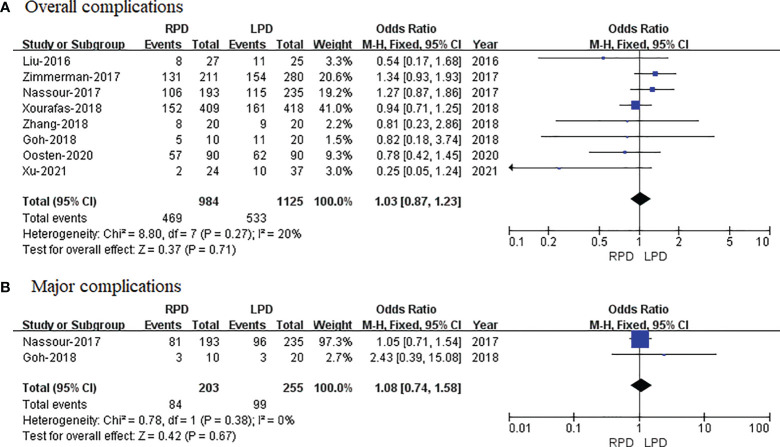
Forest plot of comparison of RPD versus LPD. **(A)** Forest plot for overall complications. **(B)** Forest plot for major complications. RPD, robotic pancreaticoduodenectomy; LPD, laparoscopic pancreaticoduodenectomy.

Eight studies that encompassed 2,109 patients (984 and 1,125 underwent RPD and LPD, respectively) reported the overall complications. Data analysis of 2,109 patients revealed that two approaches had similar overall complication rates (OR = 1.03; 95% CI 0.87 to 1.23; p = 0.71) with low heterogeneity (I^2^ = 20%) and analyzed in FEM (**Figure 4A**).

#### Major Complications

Only two studies reported the major complications. The meta-analysis showed no difference in major complications in the two groups (OR = 1.08; 95% CI 0.74 to 1.58; p = 0.67), with no heterogeneity (I^2^ = 0%) in the FEM (**Figure 4B**).

##### Postoperative Pancreatic Fistula

**Figure 5:**
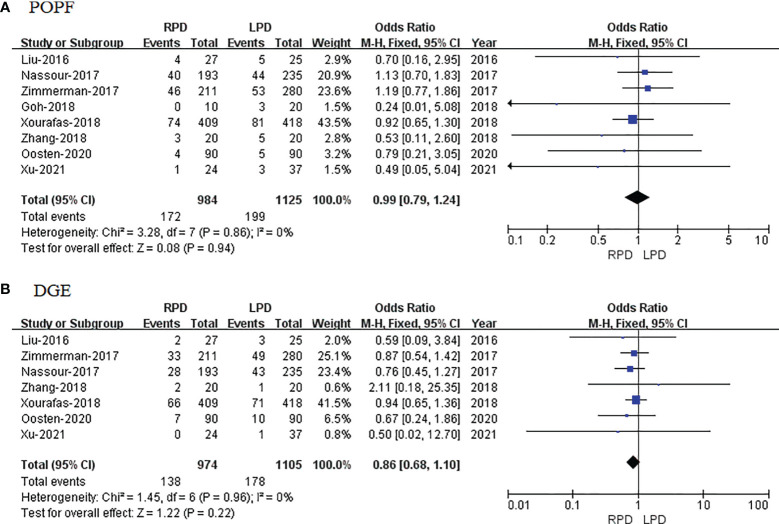
Forest plot of comparison of RPD versus LPD. **(A)** Forest plot for postoperative pancreatic fistula. **(B)** Forest plot for delayed gastric emptying. RPD, robotic pancreaticoduodenectomy; LPD, laparoscopic pancreaticoduodenectomy.

POPF data were available in 8 studies. The meta-analysis showed that RPD has similar POPF as compared with the LPD group (OR = 0.99; 95% CI 0.79 to 1.24; p = 0.94), with no heterogeneity (I^2^ = 0%) in the FEM (**Figure 5A**).

##### Delayed Gastric Emptying

DGE data were available in 7 studies. The meta-analysis showed that RPD has similar DGE as compared with the LPD group (OR = 0.86; 95% CI 0.68 to 1.10; p = 0.22), with no heterogeneity (I^2^ = 0%) in the FEM (**Figure 5B**).

#### Vascular Resection

**Figure 6:**
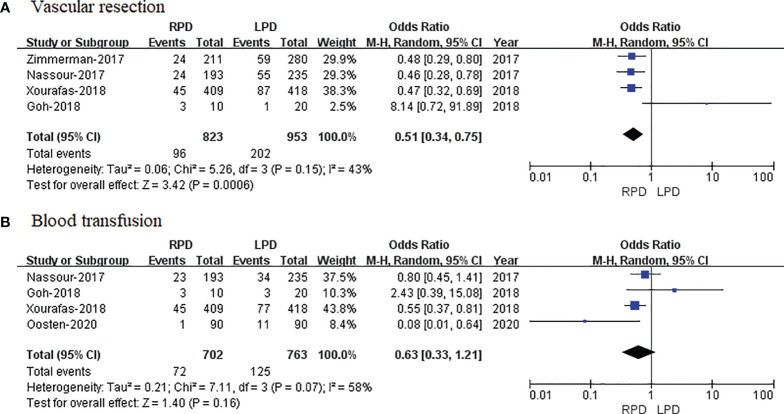
Forest plot of comparison of RPD versus LPD. **(A)** Forest plot for vascular resection. **(B)** Forest plot for blood transfusion. RPD, robotic pancreaticoduodenectomy; LPD, laparoscopic pancreaticoduodenectomy.

Four studies that included 1,776 patients (823 who underwent RPD and 953 who underwent LPD) assessed vascular resection, and the result of the meta-analysis revealed that RPD has less vascular resection than the LPD group (OR = 0.51; 95% CI 0.34 to 0.75; p = 0.0006) (**Figure 6A**).

#### Blood Transfusion

Four studies that included 1,405 patients (702 who underwent RPD and 703 who underwent LPD) assessed blood transfusion, and the result of meta-analysis revealed that RPD has similar blood transfusion as compared with the LPD group (OR = 0.63; 95% CI 0.33 to 1.21; p = 0.16) (**Figure 6B**).

#### Conversion Rate

**Figure 7:**
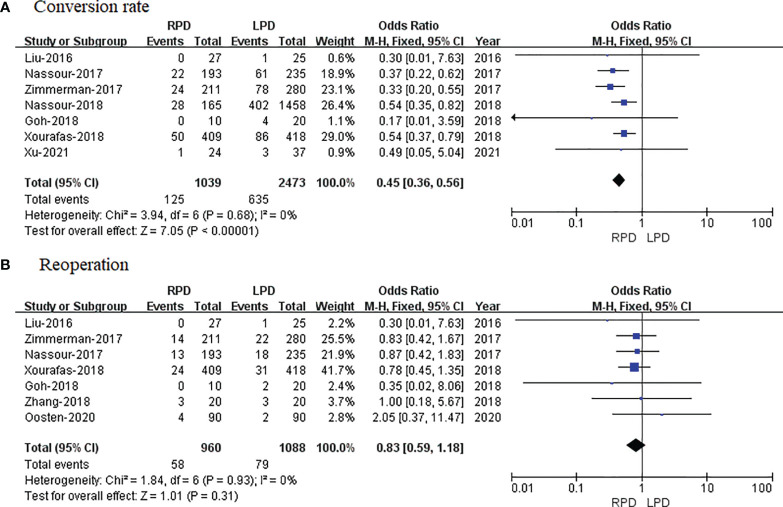
Forest plot of comparison of RPD versus LPD. **(A)** Forest plot for conversion rate. **(B)** Forest plot for reoperation. RPD, robotic pancreaticoduodenectomy; LPD, laparoscopic pancreaticoduodenectomy.

Seven studies with a total of 3,512 patients reported a conversion rate. The meta-analysis revealed that RPD presented lower conversion rate than the LPD group (OR = 0.45; 95% CI 0.36 to 0.56; p < 0.00001), with no heterogeneity (I^2^ = 0%) in the FEM (**Figure 7A**).

### Reoperation

Reoperation data were available in 7 studies. The meta-analysis indicated no significant difference in reoperation between the RPD and LPD groups (OR = 0.83; 95% CI 0.59 to 1.18; p = 0.31), with no heterogeneity (I^2^ = 0%) in the FEM (**Figure 7B**).

### Publication Bias

**Figure 8:**
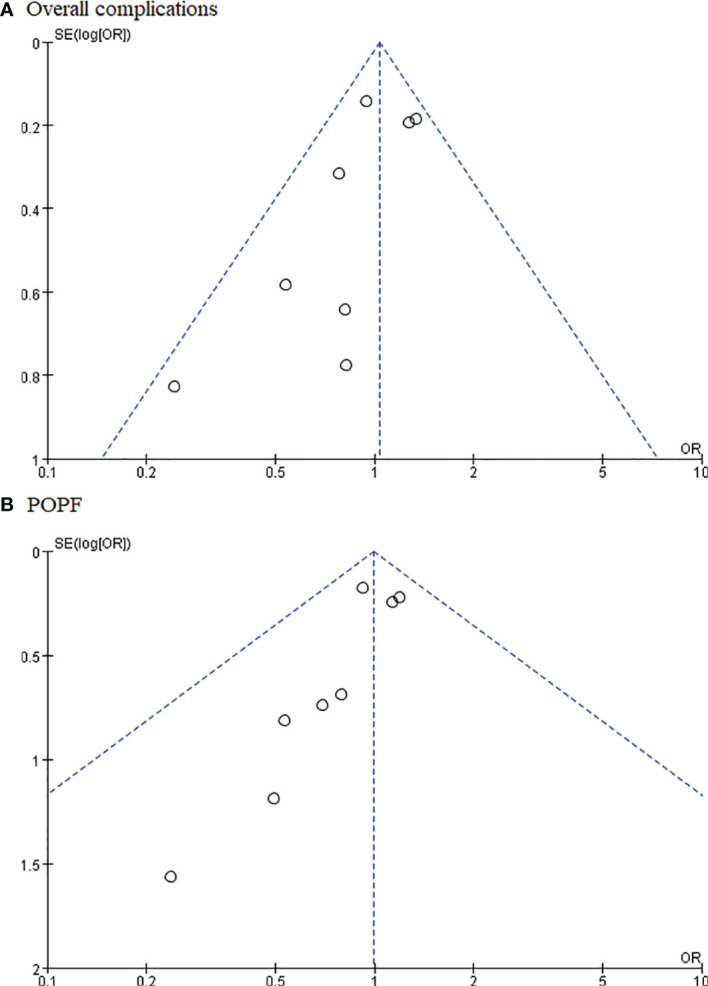
Funnel plots for **(A)** overall complications and **(B)** postoperative pancreatic fistula.

The publication bias was investigated by Begg’s funnel plot. All studies lie inside the 95% CIs in the funnel plot of overall complications and POPF, which indicated no obvious potential publication bias (**Figure 8**).

## Discussion

Minimally invasive surgery has become a worldwide trend to reduce wounds and mitigate pain. However, the role of RPD has not been well established in the era of minimally invasive surgery. Since Giulianotti et al. first performed RPD surgery in 2003 (3), with the development of robotic equipment and the accumulation of surgical experience, RPD has been gradually implemented in the field of pancreatic surgery (21). During the past decade, substantial changes have been introduced in the management of pancreatic cancer with an increased enthusiasm for minimally invasive approaches. Over the recent years, more and more studies have explored the safety and efficiency of RPD. Several studies have indicated that RPD is safe and feasible, and the curative effect is similar to that of the open approach, but it requires a higher requirement for the operator and is relatively time-consuming (5, 22). The introduction of a robotic surgical platform came with several advantages over the laparoscopic approach, yielding a better range of motion, improved ergonomics, and enhanced dexterity while allowing 3D optics. Although robotic surgery is considered to be superior to laparoscopy in several disciplines, including colorectal, gastric, pancreatic, gynecological, and urological procedures, there are some controversies about the safety and efficacy of RPD compared with LPD (23–27). In order to explore the real efficacy of RPD, this meta-analysis included relevant studies from 2016 to 2021 to explore the safety and effectiveness of RPD and LPD. All of the included studies were retrospective studies, and all of them were of relatively high quality according to NOS, as shown in **Table 1**.

Three was only one meta-analysis comparing perioperative and oncologic outcomes of RPD to LPD published (8). However, the sample size of this meta-analysis was relatively small. The study of Kamarajah et al. only focused on perioperative outcomes between RPD and LPD, and 6 articles with 3,462 patients were included. They found that RPD has similar morbidity, blood loss, operative time, shorter length of stay, lower conversion rates, and blood transfusion rates in comparison with LPD (8).

Operating time is one of the most considered surgical variables when robotic surgery is compared with laparoscopy. This meta-analysis revealed that RPD has a similar operative time compared with laparoscopy, in spite of the robotic system needing much time to set and dock. The meta-analysis showed that RPD was associated with less blood loss, which was contrary to the study of Kamarajah et al. (8). It could be explained that the robotic platform provides a magnified 3D image and eliminates hand tremor, allowing for precise suturing, better control of small blood vessel bleeding, and reduction of blood loss (13). Another reason is that patients in the RPD group are highly selected and the tumor is of early stage and/or begin. This is why RPD has less vascular resection and blood loss.

With regard to the complications, our study demonstrated no significant difference between the RPD and LPD techniques overall and regarding major complications. There is no significant difference in terms of POPF and DGE, reoperation, and blood transfusion. POPF and DGE are two main and serious technical complications and play a vital role in postoperative recovery. Our meta-analysis revealed no significant difference between the RPD and LPD. In regard to vascular resection, our study showed that RPD has evidently lower vascular resection rates than laparoscopy. The main reason is that patients for RPD are highly selected and hardly with tumor involvement of the portal vein or superior mesenteric vein. In addition, when looking at more frequent combined vascular resections in LPD, RPD is believed to be applied in well-selected and less complicated patients, leading to favorable short-term postoperative surgical outcomes. Regarding the number of LN dissections, this meta-analysis showed that RPD had more harvested LNs than LPD. It could be explained that RPD has a magnified 3D view and a tremor filter, which contribute to precise dissection and lymphadenectomy. What is more, some studies included pylorus-preserving pancreaticoduodenectomy and standard pancreaticoduodenectomy, which may determine the number of retrieved LNs due to additional LNs from omentum or peri-gastric LNs. In addition, the detail for each group that has a particular surgical approach is not reported and may represent some selection bias.

When it comes to long-term survival, to the best of our knowledge, there is still no RCT comparing the long-term survival between RPD and LPD. The largest overall survival outcome data of RPD and LPD come from the USA (17). Nassour et al. utilized the U.S. National Cancer Database, which reported 1,623 minimally invasive pancreaticoduodenectomy (165 underwent RPD and 1,425 underwent LPD) and revealed no difference in median overall survival for pancreatic adenocarcinoma between RPD and LPD (RPD 22.7 months vs. LPD 20.7 months; p = 0.445). RPD has similar 3-year overall survival rates with LPD (33% vs. 31%, p = 0.205) (17).

Although the present meta-analysis included 9 studies to draw a more convincing conclusion, there are some limitations in this study that need to be addressed. Firstly, we acknowledge that this meta-analysis does have some limitations related to possible publication bias because of the exclusion of non-English and non-Chinese articles, and patients’ demographic and comorbidity heterogeneities are high in some included studies. Detailed oncologic data such as staging of pancreatic tumor, histologic subtype, type of surgery, and tumor type are lacking in some studies. Given that these clinical factors may considerably impact outcomes and overall survival, the absence of these components should be considered as an additional limitation and a possible confounder. Secondly, only one study reported long-term survival; further, high-quality RCTs with survival outcomes are expected to assess the safety and efficiency of RPD. Additionally, all studies in this review do not stratify outcomes between benign and malignant indications, which could cause a potential source of bias and have an effect on reliable conclusions. Chronologically, RPD is the next surgical approach to LPD, which means that surgical technique and concept might be basically derived from direct and indirect experiences of LPD. Techniques for RPD might be positively modified from those of LPD. What is more, high heterogeneity among studies exists; therefore, results from meta-analysis should be taken with caution.

In conclusion, the present meta-analysis comparing RPD and LPD revealed that RPD is a safe and feasible approach. Further, large-scale and multicenter clinical RCTs are expected to assess the efficiency of RPD.

## Data Availability Statement

The original contributions presented in the study are included in the article/supplementary material. Further inquiries can be directed to the corresponding author.

## Author Contributions

Study concept and design: LO, JZ, QF, and ZZ. Acquisition of data: all authors. Analysis and interpretation of data: LO, JZ, QF, ZZ, and HM. Drafting of the manuscript: LO, JZ, QF, ZZ, and GZ). Critical revision of the manuscript for important intellectual content: GZ. Administrative, technical, or material support, and study supervision: GZ. All authors contributed to the article and approved the submitted version.

## Conflict of Interest

The authors declare that the research was conducted in the absence of any commercial or financial relationships that could be construed as a potential conflict of interest.

## Publisher’s Note

All claims expressed in this article are solely those of the authors and do not necessarily represent those of their affiliated organizations, or those of the publisher, the editors and the reviewers. Any product that may be evaluated in this article, or claim that may be made by its manufacturer, is not guaranteed or endorsed by the publisher.
